# Hippocampal subfield alterations in pediatric patients with post-traumatic stress disorder

**DOI:** 10.1093/scan/nsaa162

**Published:** 2020-12-14

**Authors:** Lei Li, Nanfang Pan, Lianqing Zhang, Su Lui, Xiaoqi Huang, Xin Xu, Song Wang, Du Lei, Lingjiang Li, Graham J Kemp, Qiyong Gong

**Affiliations:** Huaxi MR Research Center (HMRRC), Department of Radiology, West China Hospital of Sichuan University, Chengdu 610041, China; Huaxi MR Research Center (HMRRC), Department of Radiology, West China Hospital of Sichuan University, Chengdu 610041, China; Huaxi MR Research Center (HMRRC), Department of Radiology, West China Hospital of Sichuan University, Chengdu 610041, China; Huaxi MR Research Center (HMRRC), Department of Radiology, West China Hospital of Sichuan University, Chengdu 610041, China; Huaxi MR Research Center (HMRRC), Department of Radiology, West China Hospital of Sichuan University, Chengdu 610041, China; Huaxi MR Research Center (HMRRC), Department of Radiology, West China Hospital of Sichuan University, Chengdu 610041, China; Huaxi MR Research Center (HMRRC), Department of Radiology, West China Hospital of Sichuan University, Chengdu 610041, China; Department of Psychiatry and Behavioral Neuroscience, University of Cincinnati College of Medicine, Cincinnati, OH 45267, USA; Mental Health Institute, The Second Xiangya Hospital of Central South University, Changsha 410008, China; Liverpool Magnetic Resonance Imaging Centre and Institute of Life Course and Medical Sciences, University of Liverpool, Liverpool L693BX, UK; Huaxi MR Research Center (HMRRC), Department of Radiology, West China Hospital of Sichuan University, Chengdu 610041, China; Research Unit of Psychoradiology, Chinese Academy of Medical Sciences, Chengdu 610041, China

**Keywords:** stress, post-traumatic stress disorder, hippocampus, magnetic resonance imaging, psychoradiology

## Abstract

The hippocampus, a key structure with distinct subfield functions, is strongly implicated in the pathophysiology of post-traumatic stress disorder (PTSD); however, few studies of hippocampus subfields in PTSD have focused on pediatric patients. We therefore investigated the hippocampal subfield volume using an automated segmentation method and explored the subfield-centered functional connectivity aberrations related to the anatomical changes, in a homogenous population of traumatized children with and without PTSD. To investigate the potential diagnostic value in individual patients, we used a machine learning approach to identify features with significant discriminative power for diagnosis of PTSD using random forest classifiers. Compared to controls, we found significant mean volume reductions of 8.4% and 9.7% in the right presubiculum and hippocampal tail in patients, respectively. These two subfields’ volumes were the most significant contributors to group discrimination, with a mean classification accuracy of 69% and a specificity of 81%. These anatomical alterations, along with the altered functional connectivity between (pre)subiculum and inferior frontal gyrus, may underlie deficits in fear circuitry leading to dysfunction of fear extinction and episodic memory, causally important in post-traumatic symptoms such as hypervigilance and re-experience. For the first time, we suggest that hippocampal subfield volumes might be useful in discriminating traumatized children with and without PTSD.

## Introduction

Post-traumatic stress disorder (PTSD) is a debilitating psychiatric disorder characterized by re-experiencing, arousal, avoidance symptoms, and negative cognitions and emotion (American Psychiatric Association, 2013). Children and adolescents exposed to trauma in the crucial period for physical and psychological development are particularly vulnerable to developing PTSD, with potentially lifelong suffering (McLaughlin *et al.*, 2013). Pediatric PTSD is common: in a large US survey, ∼8% of adolescents exposed to traumatic experiences met the diagnostic criteria for PTSD by age 18 years (McLaughlin *et al.*, 2013). Traumatic stress in children is thought to disrupt neuroplasticity and affect the development of normal cognitive and emotional function (Lupien *et al.*, 2009; Davidson and McEwen, 2012). The key brain structure vulnerable to stress is the hippocampus, which has often been implicated in the pathophysiology of PTSD. However, despite much research on the hippocampus in adult PTSD, few studies have focused on pediatric patients and how hippocampal development may be altered.

The glucocorticoid receptor–rich hippocampal neuron is exquisitely vulnerable to stress (Sapolsky *et al.*, 1985, 2000) and plays a critical learning role in episodic memory, fear and extinction, which are important processes in the neuropathology of PTSD (Peters *et al.*, 2010; Maren *et al.*, 2013). Bremner *et al.* first found evidence for the effects of traumatic stress on the hippocampus by measuring hippocampal volume using magnetic resonance imaging (MRI) in veterans with PTSD (Bremner *et al.*, 1995). Many studies since have reported structural alterations in the hippocampus in PTSD (Geuze *et al.*, 2005; Woon *et al.*, 2010). For example, in 794 PTSD participants in a multi-site Enhancing Neuroimaging Genetics through Meta-analysis (ENIGMA) study, hippocampal volume was decreased in PTSD patients compared with trauma-exposed individuals without PTSD (‘non-PTSD’) (Logue *et al.*, 2018). While decreased hippocampal volume is commonly reported in adult PTSD patients (Kuhn and Gallinat, 2013; Li *et al.*, 2014), neuroimaging results in pediatric PTSD are inconsistent. Right hippocampal volume has been reported as decreased in maltreated youths with PTSD (Thomaes *et al.*, 2010; Morey *et al.*, 2016) and inversely correlated with PTSD symptoms (Morey *et al.*, 2016); however, other studies on pediatric PTSD have detected no significant alterations in the total hippocampal volume (Woon and Hedges, 2008; Keding and Herringa, 2015; Morey *et al.*, 2016) nor has a one-year longitudinal study (Heyn *et al.*, 2019). One possible reason is that considering the hippocampus as a whole may obscure important abnormalities in critical sub-structures.

The hippocampus is complex in structure and function: it has multiple subfields, with distinct histological characteristics, and differential vulnerabilities to stress, which may play different functional roles in PTSD (de Flores *et al.*, 2019; Fogwe and Mesfin, 2020). For example, the presubiculum and subiculum are critically involved in fear extinction function via anatomical connectivity with the dorsolateral prefrontal cortex and amygdala (Teicher *et al.*, 2012). The hippocampal tail is more relevant for spatial information and negative emotion (Strange *et al.*, 2014). The cornu ammonis (CA1–4) and the dentate gyrus (DG) are active in summarizing (pattern completion) and separating (pattern separation) sensory cues in specific contexts (Yassa and Stark, 2011), and in context-dependent memory retrieval (Knierim and Neunuebel, 2016). In the hippocampal amygdala transition area (HATA), the hippocampus is tightly co-located and interconnected with the amygdala at the cellular level, and it is involved in memory processing (Fudge *et al.*, 2012). Investigating hippocampal subfields instead of the whole hippocampus may therefore reveal more subtle, and more causally relevant, pathophysiological mechanisms in PTSD. Using high-resolution scanners and improved sequences, hippocampal subfields have been explored in PTSD using both manual (Wang *et al.*, 2010; Postel *et al.*, 2019) and automatic segmentation methods (Chalavi *et al.*, 2015; Averill *et al.*, 2017; Hayes *et al.*, 2017; Luo *et al.*, 2017; Chen *et al.*, 2018; Ahmed-Leitao *et al.*, 2019). The most common subfield alterations in patients with PTSD compared to non-PTSD controls are decreased volume of CA2–3, CA4 and DG (Wang *et al.*, 2010; Hayes *et al.*, 2017; Luo *et al.*, 2017; Chen *et al.*, 2018; Postel *et al.*, 2019); supporting the causal relevance of these changes, CA4/DG subfield volume negatively correlated with PTSD symptom severity (Hayes *et al.*, 2017). Subiculum and presubiculum volume were also decreased in PTSD patients (Chalavi *et al.*, 2015; Luo *et al.*, 2017), especially in those comorbid with dissociative symptoms (Chalavi *et al.*, 2015). Left HATA volume was decreased in patients with PTSD secondary to early childhood trauma (Ahmed-Leitao *et al.*, 2019), while in veterans with PTSD, HATA volume was negatively correlated with the Clinician-Administered PTSD Scale (CAPS) symptom severity (Averill *et al.*, 2017).

Almost all hippocampal subfield studies on PTSD have focused on adults (Wang *et al.*, 2010; Chalavi *et al.*, 2015; Averill *et al.*, 2017; Hayes *et al.*, 2017; Luo *et al.*, 2017; Chen *et al.*, 2018; Ahmed-Leitao *et al.*, 2019). The only study of adolescent patients (13–18 years old) with PTSD found decreased CA2–3/DG volume using a manual segmentation of the hippocampus into only four areas: subiculum, CA1, CA2–3/DG and hippocampal tail (Postel *et al.*, 2019). Manual segmentation, although laborious, has been regarded as the gold standard; however, it is hard to maintain good interrater reliability (Van Leemput *et al.*, 2009). With the advantages of nearly full automation, good test–retest reliability and less segmentation noise, the automated segmentation technique implemented in FreeSurfer 6.0 software can now segment the hippocampus into a larger number of subfields as accurately as the manual method (Whelan *et al.*, 2016; Cover *et al.*, 2018; Schmidt *et al.*, 2018). Other limitations of the earlier study were the inclusion of adolescent PTSD patients with a variety of trauma types (including witnessing suicide, sexual abuse and road traffic accident) and the comparison with non-traumatized healthy subjects rather than trauma-exposed controls. Different kinds of trauma may be associated with different patterns of gray matter alteration in PTSD (Meng *et al.*, 2016), and using non-traumatized controls may make it hard to determine whether alterations relate to PTSD specifically or simply to traumatic stress (Li *et al.*, 2014). Studies including subjects who experienced similar traumatic event as the control group may better clarify PTSD neurobiology.

To avoid these limitations, we set out to explore the hippocampal subfields using an automated segmentation method, in a pediatric population who all experienced a similar traumatic event, comparing those who did and did not develop PTSD. Based on the evidence that the immature hippocampus responds to early stress by releasing corticotropin-releasing hormone (Chen *et al.*, 2004) and that subfield hippocampal volumes are decreased in individuals with a history of childhood maltreatment (Andersen *et al.*, 2008; Weniger *et al.*, 2008; Mehta *et al.*, 2009; Teicher *et al.*, 2012; Chaney *et al.*, 2014), we hypothesized that specific hippocampal subfield abnormalities might be detected in the pediatric PTSD patient group relative to the non-PTSD control group. In addition, we examined the subfield-centered functional connectivity alterations to explore functional aberrations related to the anatomical change. To test the potential for clinical translation, we also performed single-subject classification using a machine learning approach; such methods, essentially multivariate pattern analyses, have emerged as a powerful tool to categorize individuals at the individual level (Lei *et al.*, 2019). We hypothesized that measures of hippocampal subfield volumes would have significant discriminative power for the diagnosis of PTSD.

## Methods

### Participants

The subjects were survivors of a magnitude 8.0 earthquake in Sichuan Province of China. The PTSD Checklist (PCL) scale (McDonald and Calhoun, 2010), a 17-item self-report measure, was used to screen survivors 8–15 months after the earthquake, and those with PCL scores >35 then undertook CAPS administered by a psychiatrist (L.L., with 32 years of experience). The Structured Clinical Interview for The Diagnostic and Statistical Manual of Mental Disorders, forth edition (DSM-IV) diagnosis was used to confirm the PTSD diagnosis. The PTSD group was all those with CAPS scores >50, while those with PCL <30 were considered to be non-PTSD controls, experiencing the same traumatic event but with no significant symptoms of PTSD.

The inclusion criteria were as follows: (i) personal experience of the earthquake, or witnessing serious injury, building collapse or death; (ii) age <18 years and (iii) intelligence quotient >80. On these criteria, a total of 260 pediatric earthquake survivors including 161 patients and 99 controls were identified. Exclusion criteria were as follows: (i) loss of consciousness >5 min, or physical injury or serious head trauma (*n* = 7); any current or past history of (ii) affective or psychotic disorder comorbidity (*n* = 42) or (iii) alcohol or drug abuse (*n* = 10); (iv) standard contraindications to MRI (*n* = 30) and (v) left-handedness (*n* = 10). For patients, those with CAPS score >30 but <50 (*n* = 10) were also excluded. All subjects included were medication-naive (24 subjects with medication use were excluded). This process yielded 28 drug-naïve, first-episode pediatric PTSD patients and 26 trauma-exposed controls who underwent MR scanning. Data from one PTSD patient were excluded because of excessive movement during acquisition and from four PTSD patients because of later segmentation failure. A diagram of patient recruitment is provided in Supplementary Figure S1. The MR data from 23 pediatric patients and 23 trauma-exposed non-PTSD controls were finally used for statistical analysis. The age range of participants was 11–16 years. This study was approved by the local research ethics committee of West China Hospital, Sichuan University. Written fully informed consent was obtained from all participants and their legal guardians prior to study participation.

### Imaging data acquisition

All subjects were scanned using a 3.0-T MRI system (Excite; GE) with an 8-channel phased-array head coil. Participants were instructed to keep their eyes closed without directed, systematic thought. The head was stabilized with cushions, and ear plugs were used. All subjects were evaluated to exclude gross brain abnormalities by an experienced neuroradiologist (L.L., 8 years of experience in neuroradiology) using conventional MRI protocols of axial T1-weighted, T2-weighted and fluid-attenuated inversion recovery images. One patient and two controls were excluded because of excessive movement during acquisition.

The 3D T1-weighted images were acquired using a single-shot spin-echo echo-planar image sequence. A whole-brain high-resolution T1-weighted image was acquired with these parameters: repetition time/echo time (TR/TE) 8.5/3.4 ms; flip angle 12°; matrix 256 × 256; field of view (FOV) 24 × 24 cm^2^; slice thickness 1 mm without gap and 156 axial slices.

The resting-state functional MRI (rs-fMRI) images were acquired using these parameters: TR/TE 2000/30ms; flip angle 90°; matrix 64 × 64; FOV 24 × 24 cm^2^; voxel size 3.75 × 3.75 × 5 mm^2^; slice thickness 5 mm without slice gap and 30 axial slices per volume.

### Volumetric analysis

Computer segmentation for anatomic T1 images was performed using FreeSurfer software 6.0 (http://surfer.nmr.mgh.harvard.edu/) and its library pipeline, ‘recon-all’. The details have been described previously (Dale *et al.*, 1999; Fischl and Dale, 2000; Fischl *et al.*, 2001, 2002, 2004a,b; Segonne *et al.*, 2004). Briefly, it includes these steps: head motion correction; skull-strip procedures; Talairach space transformation; segmentation of subcortical white matter regions and deep gray matter nuclei; signal intensity normalization and surface deformation based on intensity gradients in order to define the borders between the gray matter and white matter, and gray matter and cerebrospinal fluid.

**Fig. 1.**
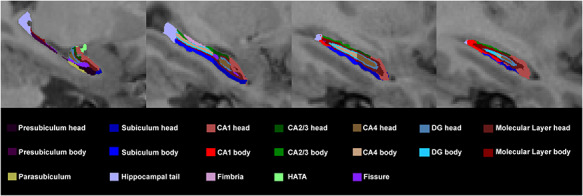
An example of segmentation of hippocampus in one subject.

Hippocampal subfields segmentation was performed using FreeSurfer 6.0 and its library function. The tetrahedral mesh-based probabilistic atlas built with ultra-high resolution *ex vivo* MRI data was employed to produce an automated segmentation of the hippocampal substructures (Iglesias *et al.*, 2015; Saygin *et al.*, 2017). By this algorithm, 12 subfield volumes were generated for the total hippocampus in each hemisphere: tail, CA1, CA2–3, CA4, subiculum, presubiculum, parasubiculum, DG, molecular layer, HATA, fimbria and fissure. An example of the segmentation for a non-PTSD subject is shown in Figure 1. Following the quality control protocol, which is similar to the ENIGMA protocol (http://enigma.ini.usc.edu/), two authors independently (L.L. and L.Z.) visually checked all the segmentations of each subject; if segmentation was judged incorrect by either, the subject was excluded (four PTSD patients and one non-PTSD control were excluded).

### Functional data preprocessing and connectivity analysis

To explore functional aberrations associated with the hippocampal subfield anatomical change, we conducted exploratory seed-based functional connectivity of the hippocampal subfield where group anatomical differences were detected. The rs-fMRI data preprocessing was performed with the Data Processing and Analysis for Brain Imaging toolkit [http://www.restfmri.net; (Yan *et al.*, 2016)], running on MATLAB R2013a. For each participant, the first 10 time points were discarded to avoid instability of the initial MRI signal. The remaining images were subjected to realignment of image and head motion correction. The motion correction strategies used the Friston 24-parameter model (Friston *et al.*, 1996; Yan *et al.*, 2013), and subjects with a motion threshold of framewise displacement (FD) >0.2 mm were excluded (Friston *et al.*, 1996). Two PTSD subjects over the motion threshold of 0.2 mm FD were excluded. Then, images were spatially normalized to the standard Montreal Neurological Institute (MNI) space echo-planar imaging template, and each voxel was resampled to 3 × 3 × 3 mm^3^. The normalized images were smoothed with 8 mm full width at half-maximum isotropic Gaussian kernel. Detrending analysis was performed to remove the effect of systematic drift or trends in rs-fMRI. To reduce the effect of the physiological artifacts, we removed several sources of nuisance signals (cerebral spinal fluid and white matter signals) from the smoothed images through linear regression, and temporal bandpass filtering (0.01–0.08 Hz) was applied.

The subfields of hippocampus in each hemisphere, comprising the CA1, CA2, CA3, DG/CA4, subicular complex (including subiculum, presubiculum and parasubiculum), HATA and entorhinal cortex, were created using probabilistic cytoarchitectonic maps of the hippocampus (Amunts *et al.*, 2005; Zilles and Amunts, 2010) included in the SPM Anatomy Toolbox [www.fz-juelich.de/inm/inm-1/DE/Forschung/_docs/SPMAnatomyToolbox/SPMAnatomyToolbox_node.html; (Eickhoff *et al.*, 2005)]. For this exploratory analysis, we only used the subfield where group anatomical differences were detected for functional connectivity analyses. Since only the right presubiculum survived the multiple comparison correction, we chose only the right presubiculum as the seed.

The seed-based resting-state functional connectivity analysis was conducted using the rs-fMRI Data Analysis Toolkit software package (http://resting-fmri.sourceforge.net) running on MATLAB R2013a. First, a seed reference time course was extracted by averaging the time courses across all voxels within each seed. Then, Pearson correlation analyses were performed between the signal average of each seed and the remainder of the whole-brain voxels in a voxel-wise manner, to generate the functional connectivity maps. Finally, Fisher’s Z transform was applied to improve the normality of the functional connectivity maps before averaging across subjects.

### Statistical analysis

Statistical analysis for demographic and clinical variables, as well as the hippocampal subfield volume was performed using SPSS software (version 16.0). Multivariate analysis of covariance was conducted to compare whole hippocampal volume and subfield volumes between groups. To address the issue of multiple comparisons, we corrected the *P* values of comparisons of all subfield volumes using the Benjamini–Hochberg false discovery rate (FDR) correction (Zhan *et al.*, 2020) in R software (version 3.5.3, http://www.rproject.com). We calculated Partial Eta Squared (η^2^) to estimate effect sizes. Age, sex, education and intracranial volume (ICV) were treated as covariates. In addition, we reported the subfield volumes with between-group changes at the nominal significance thresholds for heuristic purpose. We also examined the age/gender/education-by-diagnosis interaction effect on the volume of whole hippocampus and all the subfields.

For the functional connectivity analysis, we performed two-sample *t*-test for group comparison of *z*-value maps of the hippocampal subfield where group anatomical differences were detected in SPM8 (http://www.fl.ion.ucl.ac.uk/spm). We used a threshold adjustment method based on Monte-Carlo simulations correction for exploratory functional connectivity analysis, which is alphasim corrected with cluster size >24 (648 mm^3^), *P* <0.001, α <0.05 (http://afni.nimh.nih.gov/pub/dist/doc/manual/AlphaSim.pdf).

### Machine learning analysis

All machine learning processes were performed using the FeAture Explorer (FAE, v0.2.5, https://github.com/salan668/FAE) in Python (3.6.8, https://www.python.org/). After inputting the total and subfield volumes as features, the steps were: data normalization, data preprocessing to remove non-relevant features, feature selection and random forest modeling for classification and performance evaluation. Briefly, we first applied the normalization on the feature matrix to make the features on the same magnitude for the latter process. Next we compared the similarity of each feature pair used Pearson correlation coefficients (PCC) values, and if the PCC of the feature pair was >0.86, we removed one of them randomly. After this, the dimension of the feature space was reduced so that each feature was independent of all others. Next, we used analysis of variance to select features, and to explore the significant features corresponding to the labels. The *F*-value was calculated to evaluate the relationship between features and the label. We sorted features according to the corresponding *F*-value and selected a specific number of features to build the model. Finally, we used random forest as the classifier; this is an ensemble learning method combining multiple decision trees over different subsets of the training dataset and is an effective method to avoid over-fitting. To prove the performance of the model, we applied a stratified 5-fold cross-valuation to the dataset. This involved separating the entire dataset into five non-overlapping folds. In each iteration, four folds were used as training set and the remaining fold was used as the independent test set (from which the performance metric is calculated).

The performance of the model was evaluated using receiver operating characteristic (ROC) curve analysis, which plots a classifier’s true positive rate (sensitivity) against its false positive rate (1−specificity) as the decision threshold is varied. The area under the ROC curve (AUC) was calculated for quantification. The indices of sensitivity, specificity, accuracy, positive predictive value (PPV) and negative predictive value (NPV) were calculated in the conventional ways. To estimate statistical significance for the machine learning model, we boosted estimation 1000 times: 1000 random datasets were created by permuting the label column of the original dataset and went through the same feature selection procedure, following which we used the paired *t*-test to obtain the 95% confidence interval.

## Results

### Demographic and clinical comparisons

Table 1 summarizes the descriptive statistics for demographic and clinical characteristics in the final groups of 23 pediatric PTSD patients and 23 pediatric trauma-exposed non-PTSD controls. There were no significant differences in age, sex, education and time since trauma between the two groups.

**Table 1.** Demographic and clinical characteristics of pediatric PTSD patients and trauma-exposed control individuals without PTSD

Characteristics	PTSD (*n* = 23)	non-PTSD (*n* = 23)	*P* ^a^
Age (years)^b^	13.3 ± 1.7 (11–16)	13.0 ± 1.4 (11–16)	0.636
Education (years)^b^	8.0 ± 2.0 (6–12)	8.0 ± 2.2 (6–14)	0.944
Male/female	7/16	10/13	0.474^c^
Time since trauma (months)^b^	11.3 ± 1.6 (8–12)	11.6 ± 1.6 (10–15)	0.359
PCL	54.7 ± 3.3 (49–65)	23.2 ± 1.8 (19–27)	<0.001
CAPS	64.7 ± 4.6 (60–78)	NA	–

Unless otherwise noted, data are mean ± s.d. (range).

a Calculated by unpaired *t*-test unless otherwise noted.

b Defined at the time of magnetic resonance scanning.

c Calculated by chi-square test.

Abbreviations: CAPS, Clinician-Administered PTSD Scale; PCL, PTSD Checklist; PTSD, post-traumatic stress disorder.

### Volumetric analysis

**Fig. 2.**
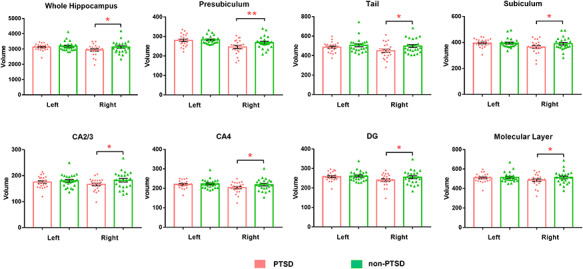
Bar charts of volumes of the hippocampal subfields in pediatric PTSD patients and trauma-exposed control individuals without PTSD. *indicates nominal significance. **indicates FDR-level significance.

The ICV values did not differ between PTSD and non-PTSD groups. Considering the whole hippocampus, the right hippocampal volume (η^2^ = 0.135, *P* = 0.017) was significantly decreased in pediatric PTSD patients compared with non-PTSD controls, but there are no differences in the left hemisphere (η^2^ = 0.027, *P* = 0.297) (see Table 2, Figure 2).

**Table 2.** Hippocampal subfield volumes (mm^3^) in pediatric PTSD patients and trauma-exposed control individuals without PTSD

Subfield region	PTSD (*n* = 23) Mean ± s.d.	Non-PTSD (*n* = 23) Mean ± s.d.	F	Partial eta squared	*P*
Left hippocampus					
Total volume	3123.4 ± 222.3	3181.1 ± 311.3	1.115	0.027	0.297
Hippocampal tail	486.4 ± 51.2	506.0 ± 72.5	2.169	0.051	0.149
CA1	589.9 ± 65.7	603.9 ± 68.0	0.517	0.013	0.476
CA2/3	175.5 ± 22.2	179.9 ± 25.2	0.639	0.016	0.429
CA4	219.9 ± 20.1	222.6 ± 23.7	0.339	0.008	0.564
Subiculum	396.0 ± 32.0	394.8 ± 37.4	0.001	<0.001	0.974
Presubiculum	280.3 ± 30.4	282.4 ± 20.9	0.572	0.014	0.454
Parasubiculum	69.1 ± 8.6	70.3 ± 9.0	0.091	0.002	0.765
Molecular layer	509.5 ± 45.1	514.5 ± 50.8	0.369	0.009	0.547
Fissure	144.0 ± 23.9	144.6 ± 16.7	0.048	0.001	0.828
Dentate gyrus	258.1 ± 23.3	260.9 ± 26.3	0.273	0.007	0.604
Fimbria	88.7 ± 11.3	91.3 ± 14.0	0.844	0.021	0.364
HATA	51.1 ± 7.1	54.3 ± 8.5	1.824	0.044	0.184
Right hippocampus					
Total volume	2936.1 ± 362.8	3125.8 ± 402.9	6.264	0.135	0.017^*^
Hippocampal tail	449.4 ± 78.9	497.7 ± 74.0	7.230	0.153	0.010^*^
CA1	588.6 ± 69.7	602.0 ± 87.0	1.171	0.028	0.286
CA2/3	166.5 ± 23.7	182.5 ± 33.3	6.382	0.138	0.016^*^
CA4	203.7 ± 28.0	218.6 ± 31.2	5.577	0.122	0.023^*^
Subiculum	365.4 ± 50.8	389.9 ± 49.7	5.645	0.124	0.022^*^
Presubiculum	246.2 ± 36.3	268.7 ± 31.7	9.861	0.198	0.003^#^
Parasubiculum	63.5 ± 11.7	67.2 ± 11.7	2.770	0.065	0.104
Molecular layer	485.0 ± 62.2	513.1 ± 67.8	4.936	0.110	0.032^*^
Fissure	144.3 ± 24.4	150.1 ± 24.2	2.412	0.057	0.128
Dentate gyrus	240.2 ± 31.8	254.9 ± 35.8	4.367	0.098	0.043^*^
Fimbria	79.0 ± 10.4	80.7 ± 11.5	0.554	0.014	0.461
HATA	48.6 ± 9.9	50.6 ± 9.0	1.391	0.034	0.245

^*^ Significant volume difference between groups at *P* < 0.05.

^#^ Significant volume difference between groups at *P* < 0.05 after Benjamini–Hochberg FDR correction. *P* values are presented before FDR correction.

Abbreviations: HATA, hippocampal amygdala transition area; PTSD, post-traumatic stress disorder.

For the subfields, pediatric PTSD patients showed volume decrease in the right presubiculum (η^2^ = 0.198, *P* = 0.003, FDR *q* = 0.036), right subiculum (η^2^ = 0.124, *P* = 0.022, FDR *q* = 0.055), right hippocampal tail (η^2^ = 0.153, *P* = 0.010, FDR *q* = 0.055), right CA2–3 (η^2^ = 0.138, *P* = 0.016, FDR *q* = 0.055), right CA4 (η^2^ = 0.122, *P* = 0.023, FDR *q* = 0.055), right molecular layer (η^2^ = 0.11, *P* = 0.032, FDR *q* = 0.064) and right DG (η^2^ = 0.098, *P* = 0.043, FDR *q* = 0.074). Only the right presubiculum volume survived multiple comparison correction. No significant differences were seen in the right CA1, parasubiculum, fimbria, fissure and HATA, or left hippocampal subfields. No significant age/gender/education-by-diagnosis interaction was found in hippocampal subfields.

### Classification performance and significantly relevant features

The model based on just two features yielded the highest AUC on the validation dataset: these two features, identified as significantly relevant, are the subfield volumes of right presubiculum and right hippocampal tail. The classification accuracy was 69% and AUC was 65%, using a repeated 5-fold cross-validation method with features from the all-relevant features selection step. The ROC curve is shown in Supplementary Figure S2. Sensitivity and specificity for discriminating pediatric patients with PTSD from non-PTSD controls were 56% and 81%, respectively, while the PPV and NPV were 75% and 65%, respectively.

### Hippocampal subfield functional connectivity

**Fig. 3.**
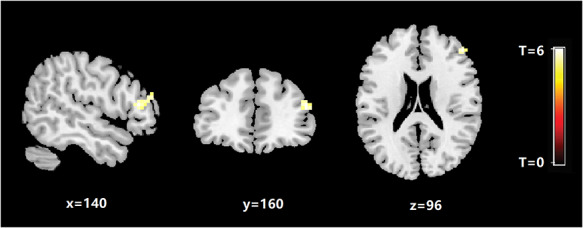
Regions showing significant group differences in the functional connectivity with the right subicular complex between pediatric PTSD patients and trauma-exposed control individuals without PTSD.

In the exploratory functional connectivity analysis, pediatric patients with PTSD showed significantly increased functional connectivity between the right subicular complex and right inferior frontal gyrus compared with non-PTSD controls (MNI coordinates: *x, y, z* = 51, 30, 12, *t* = 4.05, voxel size = 32, Figure 3). In no instance did pediatric patients with PTSD show significantly decreased subicular-complex-based functional connectivity than controls.

## Discussion

The present study explored computer-segmented hippocampal anatomic subfield abnormalities in a group of traumatized children with and without PTSD. The sample is homogeneous for surviving a single traumatic event, and all the patients are drug-naïve, which provides a good opportunity to observe disease-related changes in brain structure without confounds. We demonstrate volume reduction in specific subfields of the right hippocampus in pediatric PTSD patients relative to non-PTSD controls. The findings in right presubiculum and right hippocampal tail were the most robust results identified by machine learning. Functional connectivity analysis further revealed the functional alterations associated with the anatomical change in (pre)subiculum.

In pediatric PTSD patients compared to non-PTSD controls, we found significant volume decrease in the right presubiculum and subiculum (most prominently the presubiculum). This is consistent with a report of significant interaction effect of childhood trauma on bilateral (pre)subiculum volume (Janiri *et al.*, 2019). It is proposed that the vulnerability of the (pre)subiculum to stress may be related to its high density of glucocorticoid binding sites (Reul and de Kloet, 1985). The subiculum and presubiculum are six-layered cortical regions that lie between the hippocampus proper and the entorhinal cortex (Teicher *et al.*, 2012), and play an important role in spatial and working memory (Teicher *et al.*, 2012). It is proposed that spatial navigation and memory form the basis for other types of memory such as episodic memory (Rowland *et al.*, 2011), and episodic memory deficits are evident in PTSD, characterized by unforgettable and intrusive traumatic memories of past traumatic events that manifest as re-experiencing symptoms such as flashbacks (de Quervain, 2008). In addition, the ventral subiculum is involved in fear acquisition and extinction (O’Mara *et al.*, 2009). Specially, the presubiculum receives abundant afferents from the subiculum and sends massive projections to the medial prefrontal cortex, dorsal lateral prefrontal cortex and amygdala (Morris *et al.*, 1999; Aggleton and Christiansen, 2015), which are critical regions within the classical fear circuit (Rauch *et al.*, 2006; Rodrigues *et al.*, 2009; Parsons and Ressler, 2013). Fear extinction impairment has been proposed as the causal basis of fear-related disorders including PTSD, and pharmacological approaches that enhance fear extinction are used for PTSD treatment (Parsons and Ressler, 2013). Thus, (pre)subicular impairment may contribute to fear extinction deficit in pediatric PTSD patients.

Functional connectivity analysis identified increased connectivity between the right (pre)subiculum and right inferior frontal gyrus in pediatric PTSD patients compared to non-PTSD controls. Childhood maltreatment has been proposed to strengthen dorsal prefrontal–hippocampus connectivity in a partially compensatory way to enhance emotion regulation (Birn *et al.*, 2014). Animal studies have identified the anatomical substrate for the functional interaction between the (pre)subiculum and dorsolateral prefrontal cortex, the latter sending the fibers to connect with the presubiculum through the cingulum bundle (Goldman-Rakic *et al.*, 1984). Together with our findings, this suggests that in pediatric PTSD patients the strengthened dorsal prefrontal–(pre)subiculum pathways, relative to (pre)subicular volume decrease, may be involved, in a partially compensatory manner, in fear regulation.

Volume was also decreased in the right hippocampal tail in pediatric PTSD patients relative to non-PTSD controls. There is high variability in shape and subfield presence in the hippocampal tail, the most posterior part of the hippocampus (de Flores *et al.*, 2019), which preferentially processes spatial information and subserves visual memory (Satpute *et al.*, 2012; Poppenk *et al.*, 2013). Our result is consistent with reports of a positive correlation between hippocampal tail volume and episodic memory in children (DeMaster *et al.*, 2013, 2017). Thus, our anatomic findings in hippocampal tail may be related to episodic memory deficit in pediatric PTSD.

In addition, pediatric PTSD patients showed decreased volume in right CA 2–3, CA4, DG and molecular layer. The alterations in these subfields were more modest. Our results are consistent with a previous report of decreased CA2/DG volume in adolescent patients with PTSD relative to unexposed controls (Postel *et al.*, 2019), which was suggested to reflect a deficient ability to recovery from trauma exposure (Postel *et al.*, 2019). Pathological stress could reduce dendritic branching in the glucocorticoid receptors of CA and DG, and negatively affect neurogenesis (Leuner and Gould, 2010; Schoenfeld *et al.*, 2017). In addition, after injection of the gamma-aminobutyric acid A (GABAA) receptor antagonist picrotoxin into the prefrontal cortex, the functional connectivity between the CA3 and infralimbic prefrontal cortex was observed in adult mice along with renewal of fear memory, suggesting that this connection is involved in the regulation of extinction memory (Li *et al.*, 2018). Both CA2–3 and CA4/DG play critical roles in pattern separation, which is the ability to distinguish between similar memories in order to store them as discrete events (Yassa and Stark, 2011; Kheirbek *et al.*, 2012; Schmidt *et al.*, 2012). Thus, it may be that these alterations in CA and DG are involved in the apparition of intrusive trauma memories in pediatric PTSD.

Hippocampal volume was decreased only on the right side, perhaps reflecting lateralized hippocampal function. It is proposed that the left hippocampus is specialized for language-based memories, and the right hippocampus for spatial memory (Banks *et al.*, 2012; Kesner and Rolls, 2015). Interesting, lateralized effects have also been observed in animal studies: volume decreases of only the right CA1–3, DG and subiculum in rats exposed to 3 weeks of elevated corticosteroid (Zach *et al.*, 2010) and volume increase of only the right hippocampus with short- and long-term potentiation augmented in adulthood by neonatal exposure to novelty (a potential beneficial experience) (Verstynen *et al.*, 2001; Tang *et al.*, 2008). However, previous meta-analyses have reported decreased left total hippocampal volume in adult PTSD (Kuhn and Gallinat, 2013; Li *et al.*, 2014). Our opposite findings of right-sided deficits in subfield volume may provide a neural basis for the difference of neuropsychological profile of pediatric from adult PTSD.

Machine learning identified the right presubiculum and right hippocampal tail as significantly relevant to discriminating PTSD from non-PTSD. This means that the differentiating function obtained from these two subfield volumes is relatively stable. To our knowledge, this is the first study to use machine learning to determine whether hippocampal subfield volumes might predict PTSD diagnosis at an individual level. We found an accuracy of 69% and a relatively high specificity of 81% using the random forest model for classification and performance evaluation. Previous studies using machine learning applied to neuroimaging data to diagnose PTSD from non-PTSD controls have also achieved good results. Using whole-brain structural neuroanatomy and the support vector machine method, Gong *et al.* successfully discriminated adult PTSD patients from non-PTSD controls with 91% accuracy (Gong *et al.*, 2014b). Zhu *et al.* achieved 89% accuracy in distinguishing adult PTSD patients from non-PTSD controls using resting-state functional MRI and functional connectivity index with the relevance vector machine method (Zhu *et al.*, 2020). Using relevance vector regression, Gong *et al.* also achieved successful prediction of individual PCL scores using resting-state functional MRI data in a large group of adult survivors with and without PTSD (Gong *et al.*, 2014a). These results taken together suggest that machine learning methods have real potential to assist in diagnosis and treatment interventions for PTSD. However, it must be acknowledged that the AUC and sensitivity were relatively low. There are several possible explanations. First, the sample size is perhaps not large enough for maximal discrimination accuracy in a machine learning analysis. However, the point of this study was to use machine learning to support our main finding that the volume of hippocampal subfield (the right presubiculum) might be a useful neuroimaging biomarker to assist the clinical diagnosis of PTSD. Future studies with larger samples may help to refine the discriminating pattern. Second, only the volume of the hippocampal subfields was selected as a feature for discrimination. Despite its robustness, the sensitivity of volume quantification leaves something to be desired, as the volume often remains within the normal reference range. However, our specificity was relatively high at 81%, supporting the right presubiculum as a reliable biomarker. In future studies, other radiomic features of the hippocampal subfields, such as shape, intensity and texture, could usefully be explored as more sensitive biomarkers.

This study has some limitations. First, though the design comparing survivors with and without PTSD has advantages for identifying brain changes related to PTSD, it remains to be determined whether there are alterations induced by stress exposure *per se*. Future studies including a group of non-traumatized healthy controls would help to elucidate this. Second, whether the hippocampal subfield volume alterations we observed are pre-existing risk factors or by-products of illness remains undetermined. Longitudinal follow-up in future studies would help clarify this. Third, the hippocampal subfield template for functional connectivity analysis is not exactly the same as the template for volume analysis, partly because the lower resolution in rs-fMRI makes it harder to separate the functional image into subfields than it is with T1 images. Thus, the functional alterations do not exactly correspond to the anatomical change, and the functional connectivity analysis should therefore be considered exploratory. Fourth, the sample size was relatively small, which limited discrimination accuracy (only 69% accuracy) for the machine learning analysis. The machine learning results should therefore be considered preliminary. Future studies will need a larger sample to increase statistical power. Finally, anatomical deformation of the hippocampus may possibly decrease segmentation accuracy; however, all subjects included in the present study had no observable software failure by visual inspection.

In conclusion, our findings demonstrate hippocampal subfield volume abnormalities in a group of homogeneous single-incident traumatized children with PTSD. The most robust findings are in the right presubiculum and right hippocampal tail, which are involved in the classical fear circuit in PTSD. We suggest that altered hippocampal subfield volumes in PTSD may to some extent reflect potential deficits in fear extinction and episodic memory, causally important in post-traumatic symptoms such as hypervigilance and re-experience symptoms. Further, the anatomical alteration in the right presubiculum was associated with functional connectivity increase with the right inferior frontal gyrus. In addition, for the first time, we suggest that, using machine learning, hippocampal subfield volumes might be useful to discriminate traumatized children with PTSD from those without PTSD. Longitudinal studies investigating the interaction of genetic polymorphisms and environment, and their molecular influences on hippocampal subfield volumes, will be important to help elucidate the etiology and neurology of PTSD in the future. Finally, our study is important in “psychoradiology” (https://radiopaedia.org/articles/psychoradiology) (Gong, 2020), the application of clinical imaging to psychiatry and psychology and in guiding individual diagnostic and treatment decisions (Lui *et al.*, 2016; Kressel, 2017; Port, 2018).

## Supplementary Material

nsaa162_Supp
