# Supplementary material for: Hippocampal subfield alterations in pediatric patients with post-traumatic stress disorder
Source: Soc Cogn Affect Neurosci. 2020 Dec 14;16(3):334–44. doi: 10.1093/scan/nsaa162 (PMC7943370; doi:10.1093/scan/nsaa162)

**Supplementary Material**

**Methods**

Figure S1. Flow diagram showing the inclusion and exclusion of patients

**
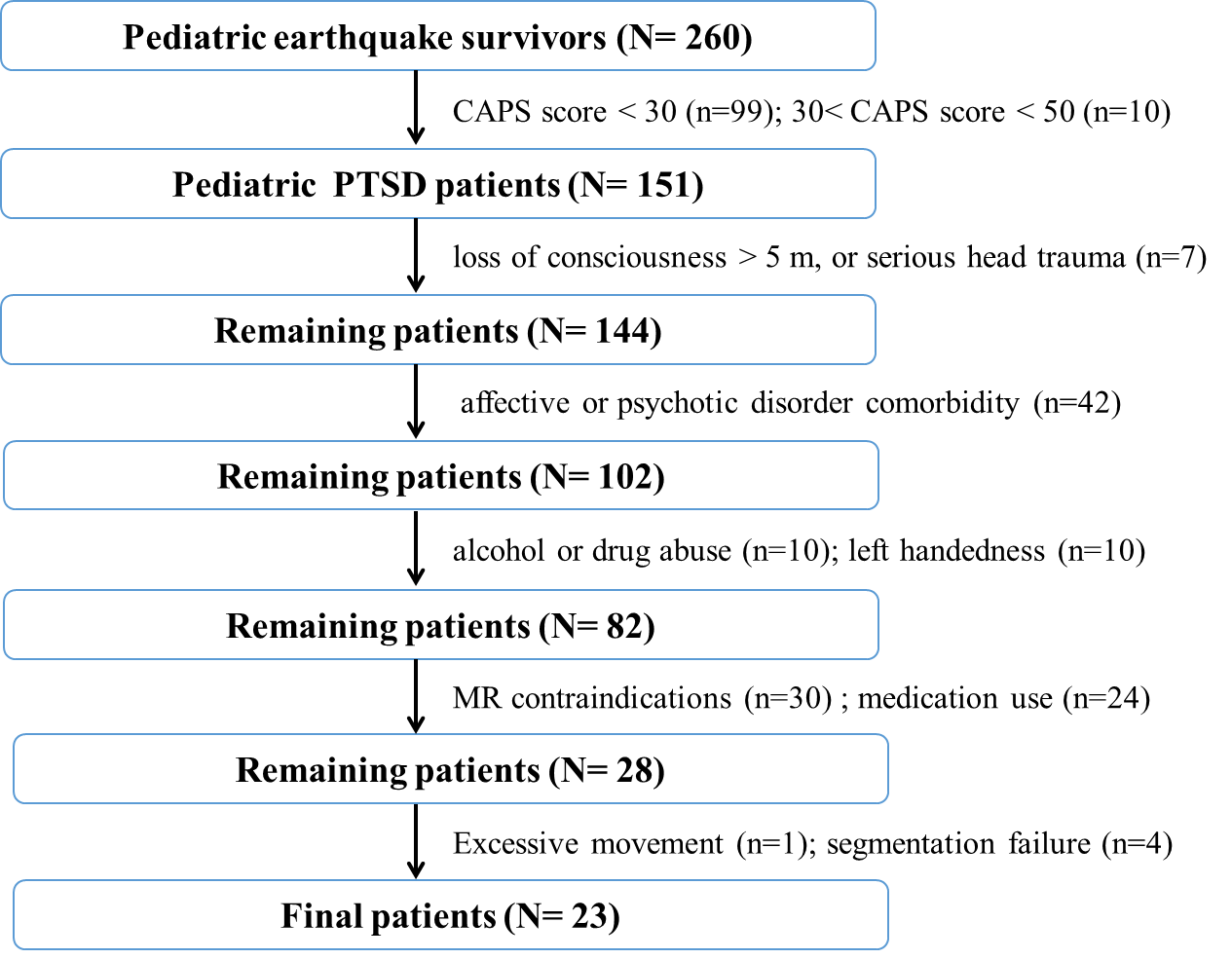
**

**Results**

Table S1. Comparison of hippocampal subfields volume between paediatric PTSD patients and non-PTSD controls by the independent two-sample t-test

|  | T value | p |
| --- | --- | --- |
| Hippocampal tail_R | -2.141 | 0.038* |
| Subiculum_R | -1.656 | 0.105 |
| Fissure_R | -0.811 | 0.422 |
| Presubiculum_R | -2.234 | 0.031* |
| CA1_R | -0.580 | 0.565 |
| Parasubiculum_R | -1.060 | 0.295 |
| Molecular layer_R | -1.464 | 0.150 |
| GC_ML_DG _R | -1.465 | 0.150 |
| CA4_R | -1.702 | 0.096 |
| Fimbria_R | -0.528 | 0.600 |
| CA3_R | -1.879 | 0.067 |
| HATA_R | -0.713 | 0.480 |

Figure S2. The Receiver Operating Characteristic curve for the performance of the hippocampal subfields in classification between paediatric PTSD patients and trauma-exposed control individuals without PTSD


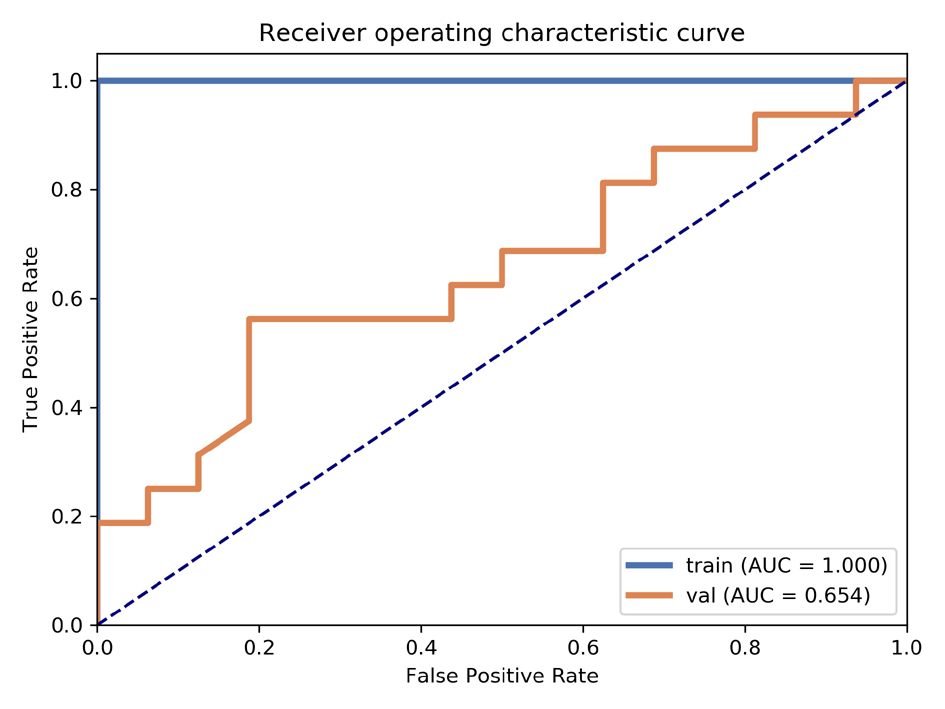

Supplement: nsaa162_Supp [file nsaa162_supp.zip › scan-20-234-File007.docx]
